# Comparing 3-month and 1-year patency rates of no-touch great saphenous vein and pedicled left internal mammary artery in off-pump coronary artery bypass grafting: a prospective non-inferiority study

**DOI:** 10.3389/fcvm.2025.1547482

**Published:** 2025-06-16

**Authors:** Teng-Yue Zhao, Bing-Jie Wang, Chu Liu, Jiang Liu, Ji-Qiang Bu, Yu Liu, Shu-Guang Zhao, Wen-Li Zhang, Zi-Ying Chen, Yu-Ming Wu

**Affiliations:** ^1^ Department of Cardiac Surgery, The Second Hospital of Hebei Medical University, Shijiazhuang, China; ^2^ Department of Physiology, Hebei Medical University, Shijiazhuang, China; ^3^ Department of Anesthesiology, The Third Hospital of Hebei Medical University, Shijiazhuang, China

**Keywords:** left internal mammary artery, non-inferiority study, no-touch great saphenous vein, off-pump coronary artery bypass grafting, rate of patency

## Abstract

**Objective:**

In this study, we compared the 3- and 12-month patency rates of great saphenous vein (GSV) grafts harvested using the no-touch technique and pedicled left internal mammary artery (LIMA) in off-pump coronary artery bypass grafting (OPCABG). We also evaluated the short-term efficacy of the grafts harvested using the no-touch technique.

**Methods:**

A non-inferiority study was conducted between June 2019 and August 2022, involving 106 patients who underwent OPCABG using the “no-touch” technique to harvest grafts from the LIMA and GSV. We aimed to assess and compare the patency rates of the pedicled LIMA graft and the no-touch GSV graft at both the 3- and 12-month postoperative intervals. Additionally, we sought to evaluate the advantages of employing the no-touch GSV graft in the context of OPCABG.

**Results:**

There was no statistically significant difference in patency between the no-touch GSV graft and the LIMA at 3 and 12 months post-OPCABG (*P* < 0.001).

**Conclusion:**

The no-touch technique can provide graft patency comparability with the LIMA; therefore, in addition to the internal mammary artery, the no-touch GSV is recommended as a graft alternative.

## 1 Introduction

Coronary artery bypass grafting (CABG) is among the most efficacious strategies for managing coronary heart disease. The surgical outcome is significantly impacted by the patency of the graft. Due to its exceptional long-term patency rate, the left internal mammary artery (LIMA) remains an unparalleled classic graft choice during CABG (1). The predominant use of the great saphenous vein (GSV) for transplantation is attributed to its superficial anatomical location, convenient accessibility, and low incidence of spasm, accounting for over 70% of all grafts. However, due to their unsatisfactory mid-long-term patency rates, conventional techniques for obtaining GSV grafts have generated controversy. A 10-year patency rate of 85% was documented by Goldman et al. for the LIMA, in contrast to only 61% for vein grafts (2). To overcome vasospasm and prevent vein wall damage, the traditional acquisition technique involves expanding and completely stripping the tissue around the GSV (3). Souza initially suggested in 1996 that no-touch technology could enhance the patency rate of the GSV (4), with its long-term patency rate being virtually equivalent to that of the LIMA (5). Numerous studies, to date, have corroborated that the no-touch technique for the GSV yields a significantly superior patency rate compared to conventional methods (6). To further highlight the benefits of the no-touch technique, a non-inferiority study was designed at a single center. The objective was to assess the short-term patency outcomes of the no-touch saphenous vein harvesting technique in this surgical procedure and compare the graft patency rate of the LIMA graft at 3 and 12 months post-off-pump coronary artery bypass grafting (OPCABG).

## 2 Methods

### 2.1 Trial design

This study was designed as a prospective, continuous, positive control, and non-inferiority study. A total of 106 patients who underwent primary isolated OPCABG at the Department of Cardiovascular Surgery, the Second Hospital of Hebei Medical University, between June 2019 and August 2022, participated in this non-inferiority clinical trial. All patients who were enrolled in the study provided informed consent, and the research was conducted after obtaining approval from the institutional ethics committee.

Attesting to the accuracy of the reported data, the first and corresponding authors assume complete responsibility for the design and execution of the study. The initial manuscript, which was finally approved by all co-authors, was drafted by the first author. Data analysis was conducted by two of the contributors. Non-authors did not make any contributions to the composition of this manuscript. There were no conflicts of interest among the research team.

### 2.2 Participants

A total of 106 consecutive enrolled patients were included in this study. A LIMA bypass was performed on the left anterior descending artery of each patient, utilizing the no-touch GSV employed as the graft vessel for the remaining target vessels. A comparative analysis of the patency rates of LIMA grafts and saphenous vein grafts was conducted within the same cohort of participants. Preoperative data were collected from the medical history and relevant preoperative examinations of each patient (Table 1).

**Table 1:** Preoperative characteristics and risk factors of patients enrolled in the study (GSV group and LIMA group).

Variables	All study patients (*n* = 106)
Age, years	60.9 ± 7.63
Female	19.8% (21/106)
Risk factors	
Body mass index	26.3 ± 2.64
Smoking	45.28% (48/106)
Hypertension	61.32% (65/106)
Diabetes mellitus	32.08% (34/106)
Dyslipidemia	
History of stroke	19.81% (21/106)
Previous PCI	3.77% (4/106)
Left main disease	66.98% (71/106)
Three-vessel disease	45.28% (48/106)
COPD	4.72% (5/106)

COPD, chronic obstructive pulmonary disease.

The exclusion criteria comprised left ventricular ejection fraction <50%, emergency surgery, preoperative intra-aortic balloon pump implantation, anticipated survival time with malignant tumor <1-year, narrow GSV (diameter <1.2 mm as visualized by vascular ultrasound), bilateral GSV varices or a prior history of vein stripping, and documented allergy to contrast media.

### 2.3 Operative strategies

**Figure 1:**
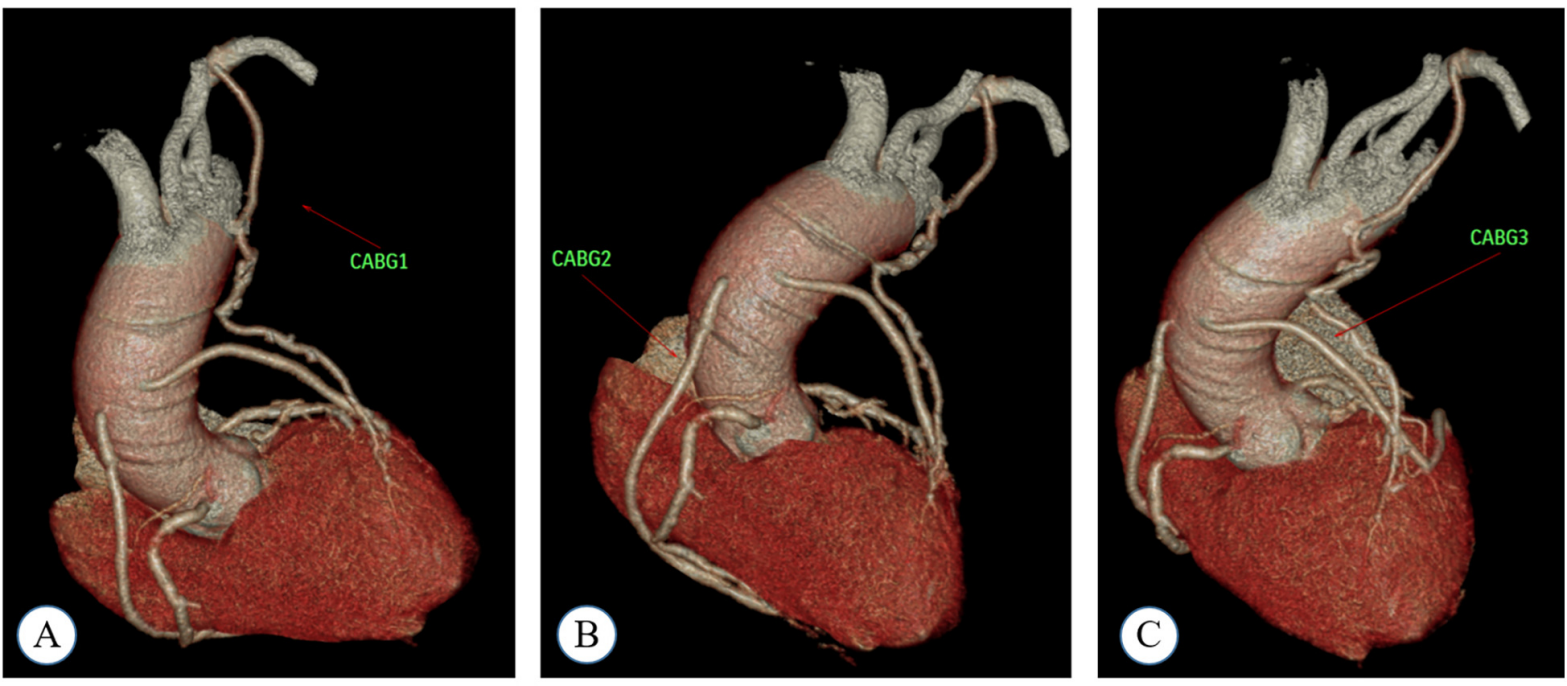
A coronary computed tomography angiography (CTA) image obtained during the postoperative follow-up of a patient who underwent coronary artery bypass grafting (CABG). In this case, the patient received a total of four grafts: LIMA-LAD, AO-SVG-PDA, and AO-SVG-DIG-OBM. Specifically, the LAD artery was targeted for CABG 1, and a LIMA graft was employed as the conduit. For CABG 2, the PDA was the target vessel, and an SVG graft was utilized. CABG 3 involved the target vessels DIG and OBM, with an SVG graft used for the bypass. **(A)** shows CABG1, **(B)** shows CABG2, and **(C)** shows CABG3.

The venous harvesting procedure was executed by a senior physician for each participant. Venous grafts were procured through bilateral longitudinal incisions on the lower legs of every patient. The adventitia and perivascular tissues of the veins were preserved, and high-pressure dilation with syringes was prohibited after the vessels were transected. A mushroom flushing needle was utilized to connect the distal end of the venous graft to a separate extracorporeal circulation machine perfusion tube (FLY, China; Stockert S3, Germany; Figure 1). In the absence of external resistance, autologous blood mixed with papaverine and heparin (30 mg papaverine and 2,500 U heparin sodium) was introduced into the blood vessel, facilitating unimpeded circulation. This process ensured the patency and absence of thrombosis of the graft. The pressure ranged from 25 to 35 mmHg. The lower leg incision was closed using two layers of continuous suture: a 3–0 intradermal suture and a continuous subcutaneous tissue 2–0 VICRYL suture. The sites with more tissue were sutured with three layers of continuous suture.

The CABG was performed by a chief physician with a minimum of 1,000 surgical procedures to their credit (7). All 106 patients underwent off-pump surgery. Dual anti-platelet therapy was administered to all patients from the day after CABG until at least 12 months after the surgery. The use of statins, antihypertensive drugs, glucose-lowering drugs, β-blockers, nitrates, and other medications was determined in accordance with the guidelines set forth by the American College of Cardiology/American Heart Association.

### 2.4 Evaluation of clinical outcomes

After the surgery, patients underwent follow-up assessments at 3 and 12 months intervals. Follow-up assessments were conducted either through outpatient visits or telephone contact; alternatives were provided if the patients were unable to attend the scheduled appointments. The primary endpoint was the assessment of arterial and venous graft occlusion at 3 and 12 months. The entire heart and grafts were scanned using a 128-slice spiral CT (Philips Brilliance ICT, Cleveland, USA). The acquired data were processed using image-related post-processing techniques, such as curved surface reconstruction and spherical display and were subsequently recorded and analyzed. Two imaging experts from our center conducted independent visual evaluations of each image. In accordance with the FitzGibbon criteria (8), graft occlusion was declared when the catheter remained completely unfilled with contrast material, or a linear sign was detected in any segment.

The secondary outcome assessed significant adverse cardiovascular and cerebrovascular events, such as cardiac death, non-fatal acute myocardial infarction, coronary revascularization, and cerebrovascular accidents, in addition to angina recurrence at 3 and 12 months post-CABG.

### 2.5 Statistical analysis

The primary endpoint was the patency rate at 3 and 12 months post-operation in order to establish the non-inferiority of the no-touch saphenous vein graft compared to the LIMA. Based on previous research (6, 7, 9), the anticipated proportions for the No-touch group were set at 96%, the anticipated proportions for the LIMA group were set at 98%, a non-inferiority boundary value of −10% was established, along with a test level *α* = 0.025 (one-sided), a test power 1-*β* = 0.80, and a sample size of 97 cases, considering a 10% loss rate, resulting in a total sample size of 106 cases.

Data analysis was conducted using SPSS 26.0 statistical software. Measurement data with a normal distribution are expressed as the mean ± standard deviation, whereas data with a non-normal distribution are expressed as the median. Frequency and percentage were used for the statistical description of count data. Patency data were analyzed using SAS 9.4 (SAS Institute Inc., Cary, NC, USA), and the formula calculation method was used to conduct the statistical analysis for the non-inferiority trial. *P* < 0.05 was considered statistically significant in this investigation.

## 3 Outcomes

### 3.1 Clinical outcomes

No perioperative deaths were recorded among the 106 patients. A solitary patient experienced postoperative gastrointestinal bleeding, which was successfully managed through emergency gastroscopy hemostasis. One patient experienced an acute myocardial infarction prior to hospitalization, requiring an emergency percutaneous coronary intervention due to recurrent coronary artery stenosis. The coronary artery graft was patent, and a stent was implanted in the right coronary artery. Postoperative atrial fibrillation developed in 9 patients (8.5%), whereas respiratory complications occurred in 3 patients.

### 3.2 Three-month angiographic results

At 3 months post-surgery, the angiographic assessment demonstrated an overall patency rate of 96.9% for the no-touch GSV and 98.1% for the LIMA (Table 2). The non-inferiority test statistic between the two groups yielded a *Z*-value of 5.155 and a *P*-value < 0.001 (Table 3). This result indicates that the patency rate of the LIMA was 98.1%, with a non-inferiority margin of −10%. The patency rates of the no-touch GSV were non-inferior to those of the LIMA. When comparing the patency of an end-to-side vein graft with that of the LIMA to eliminate any confounding effects of anastomosis, the results indicated that the patency rates of the no-touch GSV were also non-inferior to those of the LIMA (*P* < 0.001).

**Table 2:** Number of distal anastomoses between GSV and LIMA, and the conduit patency of target coronary arteries.

Target coronary arteries	Total number	Total patency rate	Number of end-to-side anastomosis	Number of end-to-side anastomosis patency rate	Side-to-side anastomosis	Side-to-side anastomosis patency rate
LAD	106	104/106 (98.1%)	106	104/106 (98.1%)	0	0
DIG	69	100%	14	100%	55	100%
RM	1	100%	0	0	1	100%
LCX	4	100%	4	100%	0	0
OBM	60	58/60 (97.0%)	54	53/54 (98.1%)	6	5/6 (83.3%)
RCA	5	100%	5	100%	0	0
PLV	46	44/46 (95.7%)	40	38/40 (95%)	6	100%
PDA	75	71/75 (94.7%)	67	64/67 (95.5%)	8	7/8 (87.5%)
Total	366	356/366 (97.3%)	290	282/290 (97.2%)	76	74/76 (97.4%)
Vein graft	260	252/260 (96.9%)	184	178/184 (96.7%)	76	74/76 (97.4%)

LAD, left anterior descending; DIG, diagonal branch; RM, ramus medianus; LCX, left circumflex; OBM, obtusemarginal; RCA, right coronary artery; PLV, posterior lateral vein; PDA, posterior descending artery.

**Table 3:** Non-inferiority test for 3-month patency rates of GSV and LIMA.

Graft	Quantity (piece)	Rate of patency	*Z*	*P*
GSV	260	96.90%	5.155	<0.001
LIMA	106	98.10%		

LIMA, left internal mammary artery.

### 3.3 Twelve-months angiographic results

At 12 months, the angiography demonstrated an overall patency rate of 96.2% for the no-touch GSV and 98.1% for the LIMA (Table 4). The non-inferiority test statistic between the two groups yielded a *Z*-value of 4.553 and a *P*-value less than 0.001 (Table 5). With a predefined non-inferiority margin of −10%, the non-inferiority test statistic for the two groups was *Z* = 4.553, *P* < 0.001, indicating that the patency rate of no-touch GSV was statistically equivalent to LIMA. In order to more clearly compare the changes of target vessel patency rate at 3 and 12 months, the summary is shown in Table 6.

**Table 4:** Twelve-month patency rates of target vessels of GSV.

Target coronary arteries	Total	Total patency rate
DIG	69	100%
RM	1	100%
LCX	4	100%
OBM	60	58/60 (97.0%)
RCA	5	100%
PLV	46	43/46 (93.5%)
PDA	75	69/75 (92.0%)

DIG, diagonal branch; RM, ramus medianus; LCX, left circumflex; OBM, obtusemarginal; RCA, right coronary artery; PLV, posterior lateral vein; PDA, posterior descending artery.

**Table 5:** Non-inferiority test for 12-month patency rates of GSV and LIMA.

Graft	Quantity (piece)	Rate of patency	*Z*	*P*
GSV	260	96.20%	4.553	<0.001
LIMA	106	98.10%		
Total	366	354/366 (96.7%)		

GSV, great saphenous vein; LIMA, left internal mammary artery.

**Table 6:** Three and twelve-month patency rates of target vessels of GSV and LIMA.

Target coronary arteries	Total	3-Month patency rate	12-Month patency rate
LAD	106	104/106 (98.1%)	104/106 (98.1%）
DIG	69	100%	100%
RM	1	100%	100%
LCX	4	100%	100%
OBM	60	58/60 (97.0%)	58/60 (97.0%)
RCA	5	100%	100%
PLV	46	44/46 (95.7%)	43/46 (93.5%)
PDA	75	71/75 (94.7%)	69/75 (92.0%)

LIMA, left internal mammary artery; DIG, diagonal branch; RM, ramus medianus; LCX, left circumflex; OBM, obtusemarginal; RCA, right coronary artery; PLV, posterior lateral vein; PDA, posterior descending artery.

## 4 Discussion

A prospective, single-blind, parallel, non-inferiority, self-controlled study was conducted to compare the patency of the no-touch saphenous vein and the pedicled LIMA during OPCABG at 3 and 12 months. The results confirmed that the patency of the saphenous-vein grafts harvested with the no-touch technique was non-inferior to that of the pedicled LIMA grafts. In this study, the 1-year patency rate of No-touch SVGs was 96.2%, which is comparable to the 18-month venous graft patency rate of 95% reported by the Souza team (5) and the patency rates observed in other No-touch studies (10).

The saphenous vein has emerged as the preferred choice for CABG due to its superficial course, ease of harvesting, and length advantage. Enhancing the patency of postoperative vascular grafts, particularly venous grafts, is of paramount importance. An investigation conducted by Souza (5) utilizing the no-touch GSV technique with an 8.5-year follow-up revealed that the long-term graft patency of the GSV obtained by the no-touch technique for CABG is comparable to that of the LIMA. Over the past decade, an increasing number of studies have corroborated that the patency of no-touch GSV grafts exhibits superior patency compared to conventional harvesting techniques. Furthermore, these studies have demonstrated that no-touch GSV grafts have a lower incidence of adverse cardiovascular events during the perioperative period (11, 12).

The high patency rate of No-touch SVG acquisition technology is widely acknowledged to be associated with its ability to emulate the patency characteristics of the left internal mammary artery (LIMA). This association is primarily manifested through the avoidance of direct contact with the vessel wall, specific endothelial protective mechanisms, and hemodynamic optimization. The technology effectively prevents vasospasm and preserves the integrity of the venous endothelium. In contrast, conventional SVG harvesting processes, which involve stripping of the adventitia and high-pressure dilation, often lead to endothelial cell detachment and inflammatory responses. The No-touch technique, however, avoids direct clamping and mechanical traction. This assertion has been corroborated by electron microscopy (13). Additionally, perivascular tissues are capable of secreting a variety of cytokines, such as adipose-derived vasodilatory factors and nitric oxide, which not only facilitate vasodilation (13) but also regulate cellular migration, proliferation, and apoptosis, thereby altering the structure of the vessel wall (14). SVGs harvested using the No-touch technique, due to the preservation of surrounding tissue support, exhibit arterial-like elastic properties post-anastomosis, thereby reducing turbulence and intimal hyperplasia caused by abnormal blood flow shear stress. Maintaining the structural integrity of the vein during surgery reduces the likelihood of graft kinking or twisting, effectively serving as a natural external scaffold.

The LIMA serves as an exemplary benchmark for assessing the effectiveness of other grafts, given its global recognition as a high-quality graft (2). The elastic lamina of the internal mammary artery is rich in elastic fibers and possesses a relatively low number of smooth muscle cells. The reduced metabolic rate of elastic fibers enhances their ischemic tolerance and diminishes their reliance on nutrient vessels. To reinforce the advantages offered by the no-touch saphenous vein as a graft material, the pedicled LIMA was utilized as the control group in this non-inferiority study. It can be regarded as the primary choice for clinical application due to its superficial course, ease of mobilization, short learning curve, and favorable length characteristics.

In contrast to the coronary artery transplantation method performed under cardiopulmonary bypass utilized by Souza et al. (15), the beating heart technique, commonly referred to as OPCABG, is primarily utilized in China. Our center has made certain modifications in continuation of the research conducted by Souza et al. and Fuwai Hospital of the Chinese Academy of Medical Sciences. Following the harvest of the no-touch GSV, its proximal end is anastomosed to the distal end of the target vessel while clamping the other end using a non-invasive bulldog clamp. After all target vessels are anastomosed, each distal end of the vein graft is sequentially connected to the aortic incision. Following the no-touch technique for GSV extraction, its distal end is attached to an independent perfusion tube on the extracorporeal circulation machine. Autologous blood containing papaverine and heparin (30 mg + 2,500 U) is smoothly injected without resistance to drain any fluid and check for the presence of blood. The required pressure ranges between 25 and 35 mmHg and depends on graft length and diameter.

In this study, the patency of end-to-side GSV graft and LIMA was tested separately to ensure control uniqueness. However, for a left anterior descending artery graft, international guidelines recommend LIMA as the preferred choice. This principle imposes limitations on target vessel selection, making it difficult to completely avoid the influence of coronary target vessels and grafts while disregarding the number of grafts. While these variables may have introduced variability to the study, it is indisputable that the results obtained from this research can be generalized to all patients undergoing CABG in accordance with this principle.

It should be noted that the present study was conducted at a single center, with a cohort of consecutive patients undergoing Off-Pump Coronary Artery Bypass Grafting (OPCABG). The selection bias inherent in a single-center study might limit the generalizability of our results to a broader patient population, as our cohort may not fully represent the diverse characteristics of patients undergoing OPCABG in other regions or institutions. While OPCABG is predominant in China, it may not be as prevalent in other regions. Therefore, the applicability of these findings to patients undergoing CABG with cardiopulmonary bypass requires further investigation. Furthermore, it is important to highlight that OPCABG and on-pump CABG involve distinct surgical procedures, patient hemodynamics, and postoperative complications. The differences in these aspects may render our findings less applicable to patients undergoing on-pump CABG, necessitating separate studies to evaluate the graft patency and outcomes in on-pump CABG populations. Lastly, although this study analyzed graft patency rates at 3 and 12 months, the 12-month data are insufficient to assess long-term patency. Comprehensive data regarding long-term patency rates can only be obtained through extended follow-up studies.

## 5 Conclusion

The results of this investigation revealed that the patency rate of saphenous vein grafts obtained using the no-touch technique is non-inferior to that of pedicled LIMA grafts, thereby confirming the comparable graft patency between the no-touch technique and LIMA. Consequently, the use of a no-touch GSV as a primary choice for grafting alongside the LIMA is recommended.

## Data Availability

The original contributions presented in the study are included in the article/Supplementary Material, further inquiries can be directed to the corresponding authors.
